# Perceived academic achievement and school commitment in the context of school sports: A qualitative study based on students’, teachers’, and parents’ perspectives

**DOI:** 10.1371/journal.pone.0351877

**Published:** 2026-06-29

**Authors:** Zeki Taş, Songül Baykara, Yılmaz Ünlü, Ayşe Önal, Ahmet Naci Dilek, Murat Şen

**Affiliations:** 1 Faculty of Sport Sciences, Physical Education and Sports Teaching Department, Sakarya of Applied Sciences University, Sakarya, Türkiye; 2 Department of Physical Education and Sports Teaching, Sakarya of Applied Sciences University, Graduate Education Institute, Sakarya, Türkiye; 3 Faculty of Sport Sciences, Sport Management Department, Bolu Abant İzzet Baysal University, Bolu, Türkiye; 4 Faculty of Sport Sciences, Department of Recreation, Afyon Kocatepe University, Afyonkarahisar, Turkey; 5 Faculty of Sport Sciences, Department of Recreation, Bartın University, Bartın, Türkiye; 6 Faculty of Sport Sciences, Department of Recreation, Sakarya of Applied Sciences University, Sakarya, Türkiye; University of Salerno: Universita degli Studi di Salerno, ITALY

## Abstract

School sports are widely associated with students’ academic and psychosocial development, yet their educational value cannot be fully understood through outcomes alone. How sports participation shapes academic achievement and school commitment depends on how it is experienced and interpreted by students, teachers, and parents. Examining these stakeholder perspectives is essential for understanding the perceived academic implications of school sports and their role within everyday school contexts. This study adopted a qualitative descriptive research design to explore perceptions of school sports and their perceived influence on academic achievement and school commitment. The study group consisted of 18 participants, including 6 high school students (grades 10–12), 6 parents whose children participated in school sports, and 6 physical education teachers working at a public high school in Sakarya, Türkiye. Participants were selected using criterion sampling. Data were collected through face-to-face focus group interviews conducted separately with each stakeholder group. The data were analyzed using descriptive qualitative analysis supported by inductive content analysis to identify recurring patterns and themes related to participants’ views and experiences of school sports. The findings indicate that school sports are perceived to positively influence students’ academic achievement and school commitment, particularly through increased motivation, concentration, and engagement with school life. Participants emphasized that sports participation strengthens emotional and behavioral commitment by enhancing feelings of belonging and active involvement in school activities. Teachers’ guidance and parents’ supportive attitudes were identified as key contextual factors, while students’ personal interest in sports shaped whether participation was experienced as supportive or demanding. This study concludes that school sports play a meaningful role in supporting students’ perceived academic achievement and school commitment when embedded within supportive school and family contexts. The findings highlight that the educational value of sports is shaped not only by participation itself but also by students’ experiences, teachers’ guidance, and parental support. By integrating multiple stakeholder perspectives, the study underscores the importance of aligning school sports practices with academic goals to foster sustained engagement and positive educational experiences.

## Introduction

Schools serve as a primary context for the development of children and adolescents, where academic learning is intertwined with social interaction, emotional growth, and identity formation. Beyond their instructional function, schools structure students’ daily experiences and contribute to shaping their engagement with learning and achievement. In this regard, students’ academic performance and their psychological connection to school are influenced by the broader educational environment and the opportunities it affords [1]. Within this context, school-based sports constitute an important component of students’ overall school experience. Participation in organized sports has been associated with various developmental outcomes, including improved self-regulation, cooperation, and social integration [2,3]. Rather than being limited to physical activity, school sports provide structured environments that may support students’ motivation, enhance time management skills, and foster a sense of belonging through interactions with peers outside the classroom [4].

Despite these commonly held assumptions, empirical findings on the relationship between school sports and academic outcomes are not entirely consistent. While a number of quantitative studies report positive associations between sports participation and academic performance, others point to potential challenges such as fatigue, increased time demands, and academic pressure [5,6]. These mixed findings indicate that the educational implications of school sports may not be adequately captured through participation rates or outcome-based measures alone. Accordingly, a more nuanced understanding is needed by examining how school sports are experienced and interpreted by students, teachers, and parents who are directly involved in the educational process.

In the present study, academic achievement is not conceptualized solely as an objective indicator such as grades or standardized test scores. Instead, it is understood as participants’ perceptions of how school sports influence students’ academic functioning, including motivation, concentration, learning engagement, and the ability to meet academic expectations. This perspective is consistent with qualitative research approaches, particularly phenomenological inquiry, which seeks to capture how individuals experience and make meaning of a given phenomenon from their own perspectives rather than relying on externally imposed measurements [7]. By focusing on perceived academic achievement, the study aims to provide a nuanced understanding of how students, teachers, and parents interpret the academic implications of school sports within the context of everyday school life.

To ensure conceptual coherence, the present study is grounded in school engagement theory, particularly the multidimensional framework proposed by Fredricks, Blumenfeld, and Paris [8]. This framework conceptualizes school engagement as a dynamic and multifaceted construct consisting of three interrelated dimensions: emotional, behavioral, and cognitive engagement. Emotional engagement refers to students’ sense of belonging, attachment, and perceived value within the school environment. Behavioral engagement encompasses observable participation in academic and extracurricular activities, attendance, and adherence to school norms. Cognitive engagement reflects students’ psychological investment in learning, their willingness to exert effort, and the extent to which they adopt and internalize academic goals and values. In the context of this study, this framework provides a useful lens for understanding how involvement in school sports may relate to different dimensions of students’ engagement with school.

This framework offers a coherent lens for examining school sports, as participation in sports may shape each dimension of school engagement in distinct yet interrelated ways. For instance, representing a school team may enhance emotional engagement by strengthening students’ identification with the school, support behavioral engagement through sustained participation in school-related activities, and foster cognitive engagement by encouraging goal orientation and persistence. In this study, school engagement is treated as the primary analytical construct, rather than using related concepts such as engagement, belonging, and attachment interchangeably. This approach contributes to conceptual clarity and ensures consistency between the theoretical framework, analytical process, and interpretation of findings.

Existing research on school sports and school engagement has predominantly relied on quantitative designs and has largely focused on students as the primary source of data [9,4]. While these studies provide valuable insights into general patterns and relationships, they offer limited understanding of how the meanings attributed to school sports are constructed and experienced within everyday school contexts. Furthermore, the perspectives of teachers and parents-who play critical roles in organizing sports activities, monitoring academic demands, and supporting students-have been comparatively underrepresented. This limitation highlights the need for research that incorporates multiple stakeholder perspectives in order to capture a more comprehensive and contextually grounded understanding of the educational role of school sports.

This limitation is not only methodological but also theoretical in nature. School commitment is widely understood as a contextual and relational construct that emerges through interactions among students, educators, families, and institutional practices [10]. Focusing exclusively on students’ perspectives may therefore oversimplify this process and obscure how meanings attributed to school sports are constructed, negotiated, and reproduced within the school context. In contrast, incorporating the perspectives of students, teachers, and parents enables a more comprehensive understanding of how school sports are interpreted and valued within the school community. The added value of a multi-stakeholder qualitative approach extends beyond the inclusion of multiple viewpoints; it lies in identifying both convergences and divergences across stakeholder groups. Examining these patterns makes it possible to better understand how expectations related to academic achievement and school commitment are formed, sustained, or contested. Such an approach contributes to refining theoretical discussions on school commitment and provides practical insights for designing school policies that integrate sports activities in ways that support rather than hinder academic engagement.

Although school sports are frequently promoted in the literature as mechanisms for enhancing discipline, belonging, and academic motivation [e.g., 4,5,11], there remains limited qualitative evidence explaining how these outcomes are perceived by students, teachers, and parents within the same educational context. In particular, descriptive research that systematically documents participants’ views on both the perceived benefits and challenges of school sports remains scarce.

Accordingly, the present study aims to explore the perceived effects of school sports on students’ academic achievement and school commitment from the perspectives and lived experiences of students, teachers, and parents. The study adopts a descriptive qualitative research design and utilizes focus group interviews to systematically elicit participants’ viewpoints. Data are analyzed through descriptive analysis supported by inductive content analysis, enabling a structured yet flexible examination of the data. This approach allows for a detailed and contextually grounded account of how school sports are understood to influence students’ academic engagement and their sense of connection to the school, while also capturing both shared patterns and variations across stakeholder perspectives.

## Methods

### Study design

This study adopted a qualitative descriptive research design to examine the perceived effects of school sports on academic achievement and school commitment based on the views and experiences of students, physical education and sports teachers, and parents. Qualitative descriptive research aims to provide a comprehensive and straightforward description of participants’ perceptions and experiences in their own terms, without imposing highly abstract theoretical interpretations [12,13]. The qualitative descriptive approach was considered appropriate for this study because the primary aim was not to uncover the essence of lived experience or to generate theory, but rather to describe and summarize how different stakeholder groups perceive and interpret the role of school sports in relation to academic engagement and school commitment. In this respect, the study focuses on presenting participants’ accounts in a clear, contextualized, and data-near manner.

Data were collected through focus group interviews conducted separately with students, physical education and sports teachers, and parents. Focus groups were chosen as they enable participants to express their views through interaction, stimulate reflection, and reveal shared as well as divergent perspectives on a common topic [14,15]. Conducting separate focus groups for each stakeholder category allowed the study to capture multiple viewpoints while maintaining clarity and coherence in the description of findings. The analytic orientation of the study was descriptive and thematic. Participants’ statements were systematically examined to identify recurring patterns and themes reflecting perceived effects of school sports on academic achievement and school commitment. Rather than aiming for generalization or theory development, the study seeks to provide a detailed, transparent, and context-sensitive account of the findings within the boundaries of the research setting [16].

### Research group

The demographic characteristics of the students participating in the study are presented in Table 1, including gender, age, and sport branch.

**Table 1:** Demographic information of students participating in research.

Participant	Gender	Age	Sport Branch
P1	Male	17	Football
P2	Male	16	Basketball
P3	Female	16	Volleyball
P4	Male	16	Athletics
P5	Female	16	Archery
P6	Female	16	Badminton

The demographic characteristics of physical education and sports teachers participating in the research are summarized in Table 2. Table 3 shows the gender and age information of the teachers.

**Table 2:** Demographic information of physical education and sports teachers participating in research.

Participant	Gender	Age
P7	Female	51
P8	Male	60
P9	Male	38
P10	Female	43
P11	Male	40
P12	Male	42

**Table 3:** Demographic information of parents participating in research.

Participant	Gender	Academic Field	Age
P13	Male	Mathematics	52
P14	Male	History	54
P15	Female	Physics	44
P16	Female	Physical education	44
P17	Female	Theology	40
P18	Female	Foreign language	46

The demographic characteristics of the students participating in the research are summarized in Table 3. Table 3 shows the gender and age information of the participating parents.

Criterion sampling, a purposeful sampling strategy, was employed to identify participants who could provide rich and relevant insights into the research problem [7]. The study was conducted in collaboration with the administration of a public Anatolian High School located in Sakarya during the fall semester of the 2024–2025 academic year. Prior to participant recruitment, institutional permission was obtained from the school administration.

Potential participants were identified with the assistance of school administrators and physical education teachers based on the predefined inclusion criteria. These criteria included: (a) students who had been actively participating in school sports for at least one year, (b) parents who closely observed and supported their children’s involvement in school sports, and (c) physical education and sports teachers who were directly involved in organizing, supervising, and observing students’ sports activities.

Following this identification process, eligible participants were approached and invited to participate in the study on a voluntary basis. All participants were informed about the purpose, scope, and procedures of the research prior to data collection, and written informed consent was obtained from all participants. Participation was entirely voluntary, and no incentives were offered. None of the invited participants declined participation or withdrew from the study.

To ensure diversity and enhance the richness of the data, variation was sought within each stakeholder group in terms of gender, age, sports branch and professional experience. The final study group consisted of 18 participants, including six student athletes, six parents, and six physical education and sports teachers. Data collection continued until thematic saturation was reached, meaning that no new themes or meaningful insights emerged from the data. The sample size of six participants per stakeholder group was considered sufficient to achieve saturation, as participants provided repeated and consistent patterns of responses across interviews. This approach is consistent with qualitative research standards, where sample size is determined by information richness rather than statistical representativeness [17,18]. In line with qualitative research standards, the size of each stakeholder group (n = 6) was considered appropriate to facilitate interaction among participants while allowing each individual sufficient opportunity to express their views in depth [15].

Although the sampling strategy aimed to include information-rich and diverse participants, the researchers acknowledge that the study was conducted within a single institutional and socio-cultural context. Therefore, the findings are not intended for statistical generalization but rather for analytical generalization and contextual transferability, supported by detailed descriptions of the research setting and participant characteristics [19].

### Data collection tool

Data were collected using a semi-structured focus group interview guide developed by the researchers. The development of the interview guide was informed by an extensive review of the relevant literature on the effects of school sports on academic achievement and school commitment (e.g., [9,16,20–27]). This review enabled the identification of key conceptual domains and recurring issues addressed in previous qualitative and mixed-methods studies.

The interview questions were designed to align with the descriptive qualitative research approach, aiming to elicit participants’ perceptions, experiences, and interpretations in a clear and accessible manner. Questions were formulated using open-ended language, arranged in a logical sequence from general to more specific issues, and carefully worded to avoid leading or suggestive phrasing. In addition to the main questions, non-directive probing prompts (e.g., “why,” “how,” “can you explain further?”) were used during the interviews to encourage participants to elaborate on their responses and to capture diverse viewpoints and experiences.

To enhance content validity, the draft interview guide was reviewed by three academic experts with experience in physical education, sport sciences, and qualitative research methodology. Based on their feedback, revisions were made to improve clarity, relevance, and alignment with the study’s research aims. Following this expert review, a pilot study was conducted with three participants who met the inclusion criteria but were not included in the main study sample. The pilot interviews allowed the researchers to assess the comprehensibility, flow, and practical applicability of the questions. To enhance transparency, examples of the interview questions and probing strategies are provided. An example of a main interview question is: “How does participation in school sports influence students’ academic achievement?” During the pilot study, this question was refined to ensure clarity and accessibility for all participant groups. For instance, more general wording was preferred over technical terminology to facilitate understanding.

In practice, non-directive probing questions were actively used to deepen participants’ responses. Examples of such probes include: “Can you explain this in more detail?”, “Why do you think this happens?”, and “Can you give an example from your experience?”. These prompts enabled participants to elaborate on their views and contributed to the emergence of rich and nuanced data.

The data obtained from the pilot study were preliminarily examined, and the interview guide was further refined in consultation with a field expert to ensure that the questions effectively captured participants’ experiences and perspectives. After these revisions, the interview guide was finalized and used consistently across all focus group interviews. The finalized interview questions are presented below.

#### Data collection context.

Data were collected through focus group interviews, a qualitative data collection method widely used to obtain in-depth and detailed information about participants’ perspectives, perceptions, attitudes, experiences, thoughts, and feelings regarding a specific phenomenon [7,15,28–31]. Focus group interviews enable participants to articulate their views through interaction, stimulate reflection, and reveal both shared and divergent perspectives on a common topic [15]. According to Bowling [28], focus group interviews facilitate the generation of in-depth knowledge through the dynamic interaction between a small group of participants and a moderator. In line with the core characteristics of qualitative research, participants’ knowledge, experiences, emotions, and interpretations are central to understanding the phenomenon under investigation [16]. Creating an environment in which participants can freely express their views therefore constitutes a key component of the focus group method.

Within the scope of the study, focus group interviews were conducted with three distinct stakeholder groups—students, teachers, and parents—comprising a total of 18 participants. Separate focus groups were organized for each group to reduce potential power imbalances and to enable participants to express their views more openly within relatively homogeneous peer settings [15]. This approach was considered appropriate for capturing not only students’ experiences but also the perspectives of parents and teachers who continuously observe students’ academic performance and school engagement, thereby contributing to a more comprehensive and descriptive understanding of the phenomenon.

Each focus group consisted of six participants, a group size considered suitable for encouraging interaction while allowing sufficient opportunity for all participants to express their views in depth [15,29]. In qualitative research, the number of focus groups is determined by the depth of information and the principle of data saturation rather than numerical representativeness. In the present study, conducting a single focus group for each stakeholder category was considered sufficient, as participants within each group shared similar contextual characteristics and provided repetitive and consistent patterns of responses during the discussions. Moreover, the relatively homogeneous composition of each stakeholder group facilitated meaningful interaction and enabled the exploration of shared and divergent views within a single session. Methodological literature supports the use of small, well-structured focus groups when participants belong to similar populations and the discussion is effectively guided, indicating that a single focus group can yield rich and sufficient qualitative data under such conditions [32,33]. All focus group interviews were moderated by the first author, who has an academic background in physical education and sports sciences and prior experience in qualitative research. The researcher did not hold any administrative or instructional role at the participating school, thereby minimizing potential power relationships with participants. Throughout the data collection process, the researcher adopted a facilitative and reflexive role, encouraged balanced participation, and remained attentive to potential biases arising from prior professional knowledge of the field [16]. In addition, reflexivity was maintained throughout the research process to minimize potential researcher bias. The moderator remained aware of their own professional background in physical education and its possible influence on data interpretation. To address this, a conscious effort was made to adopt a neutral and non-judgmental stance during the interviews, allowing participants to express their views freely without influence. Reflective notes were taken after each focus group session to critically examine the researcher’s role, assumptions, and potential biases.

Group dynamics were actively managed during the focus group sessions. Participants were encouraged to share their views openly, while care was taken to prevent dominant voices from overshadowing others. Probing questions were used to invite quieter participants to contribute, ensuring that a broad range of perspectives was represented within each group [29].

All interviews were conducted in Turkish, the native language of the participants, to allow for the most accurate and nuanced expression of experiences. Audio recordings were transcribed verbatim. When excerpts were translated into English for reporting purposes, particular attention was paid to preserving meaning by systematically comparing translations with the original transcripts and reviewing them for conceptual consistency [34]. Prior to data collection, participants were informed about the purpose of the study and provided informed consent. Confidentiality and anonymity were ensured throughout the research process.

The time and place of the interviews were determined in consideration of participants’ availability. To ensure high-quality audio recordings and participant comfort, the interview environment was arranged to be adequately ventilated, with appropriate temperature and seating conditions. Each focus group interview lasted approximately 45–60 minutes. The interviews with the student group were conducted on November 26, 2024, followed by the teacher group on December 4, 2024, and the parent group on December 7, 2024. Throughout the data collection process, methodological rigor was prioritized over logistical considerations, with particular attention given to creating a respectful and supportive environment conducive to open discussion.

### Ethics statement

This study was reviewed and approved by the Sakarya University of Applied Sciences Ethics Committee (Decision No: 50/22; Approval Date: 15.11.2024; Reference No: E-26428519-050.99-149279). All procedures were conducted in accordance with the ethical standards of the institutional and/or national research committee and with the 1964 Helsinki Declaration and its later amendments or comparable ethical standards.

Informed consent was obtained from all participants prior to their inclusion in the study. Participants were informed about the purpose of the research, the voluntary nature of participation, confidentiality of their responses, and their right to withdraw from the study at any time without any consequences. Written/oral informed consent was secured prior to data collection

### Analysis of data

Data analysis was conducted using a descriptive qualitative analysis framework, supported by inductive content analysis, in line with the aims of the study and the nature of the data obtained from focus group interviews [15,16]. This combined approach enabled a systematic description of participants’ views while also allowing for the identification of recurring patterns and meanings across the dataset.

All audio-recorded focus group interviews were transcribed verbatim. The analysis process began with repeated readings of the transcripts to achieve familiarization with the data and to gain a holistic understanding of participants’ experiences and perspectives. During this phase, initial notes were taken to capture salient ideas related to academic achievement, school commitment, and extracurricular life.

In the first stage, descriptive analysis was applied. The data were organized according to the main interview questions, which served as an initial analytic framework. Participants’ statements were summarized and described under these broad dimensions, and representative direct quotations were identified to preserve the authenticity of participants’ voices. This stage focused on presenting the data in a clear and accessible manner without imposing premature interpretation.

In the second stage, inductive content analysis was employed to move beyond description and to identify meaningful patterns within the data [7]. Coding was conducted inductively, allowing codes to emerge from the data rather than being imposed a priori. Initial codes were generated by the first author through line-by-line coding of the transcripts. These codes were then reviewed, compared, and grouped into categories based on conceptual similarity. Subsequently, higher-order themes were developed by examining relationships among categories and by refining them to ensure internal coherence and conceptual clarity.

To enhance analytic rigor, the coding scheme and emerging themes were discussed with a second researcher experienced in qualitative research. Discrepancies in coding and theme interpretation were resolved through discussion and consensus, contributing to the dependability and confirmability of the analysis. An audit trail documenting analytic decisions, code definitions, and theme development was maintained throughout the process.

Trustworthiness of the study was addressed using multiple strategies [19]. Credibility was enhanced through prolonged engagement with the data and the use of rich, direct quotations. Peer debriefing was conducted during the analysis phase to critically examine interpretations and reduce researcher bias. Dependability was supported by providing a transparent and systematic account of the analytic procedures, while confirmability was strengthened through reflexive consideration of the researcher’s role and assumptions. To support transferability, detailed descriptions of the research context, participants, and data collection procedures were provided, allowing readers to assess the applicability of the findings to other settings.

The development of themes followed a systematic inductive process progressing from raw data to codes, categories, and higher-order themes. Initially, participants’ statements were examined and segmented into meaningful units, which were labeled as initial codes. These codes were then grouped into categories based on conceptual similarity. Subsequently, broader themes were constructed by identifying patterns and relationships across categories. To enhance transparency, a coding tree illustrating this analytical progression from raw data to themes is presented (see Table 4).

**Table 4:** Coding tree: From raw data to themes.

Raw data (sample quotations)	Code	Category	Theme
“Being involved in sports makes me more disciplined in my lessons.” (P1)	Academic discipline	Learning-related attitudes	Academic motivation
“Students… are more motivated in the classroom.” (P9)	Academic motivation	Engagement in learning	Academic motivation
“I have to organize my day carefully because of training.” (P2)	Daily planning	Time organization	Time management
“It becomes difficult to balance schoolwork and sports.” (P12)	Difficulty in balance	Academic–sport conflict	Time management
“Representing my school makes me feel proud.” (P3)	School pride	Sense of belonging	School commitment
“Students feel more attached to the school.” (P10)	School attachment	School identification	School commitment
“Sports help me feel more confident.” (P5)	Increased self-confidence	Personal development	Psychosocial development
“My child has become more social.” (P16)	Socialization	Social development	Psychosocial development
“Sports may cause fatigue and affect performance.” (P13)	Fatigue	Perceived academic risks	Perceived academic risk
“I worry about missing lessons.” (P12)	Concern about missing classes	Academic concerns	Perceived academic risk
“Guiding students is essential.” (P11)	Teacher guidance	Adult support mechanisms	Adult support
“We ensure balance between school and sports.” (P17)	Parental monitoring	Family support	Adult support

### Findings

The themes and sub-themes obtained from the analysis are presented in Table 5. In addition, in order to more clearly and directly reflect the participants’ views, the explanations related to the relevant themes and the participants’ statements are provided in detail below.”

**Table 5:** Themes and sub-themes related to the effects of school sports on academic achievement and school commitment.

Theme	Sub-Themes	Relevant Stakeholders
Academic Motivation	Focus on lessons, goal setting, learning engagement	Students, Teachers
Time Management	Discipline development, balancing academic and athletic demands	Students, Parents
School Commitment	Sense of belonging, representing the school	Students, Teachers
Psychosocial Development	Self-confidence, social skills, stress management	All stakeholders
Perceived Academic Risk	Fatigue, concern about missing classes	Parents
Adult Support	Teacher guidance, parental attitudes	Teachers, Parents

In this context, regarding the theme of academic motivation, P1: *“Being involved in sports makes me more disciplined in my lessons. I try to plan my time better and focus more on my schoolwork.”*; P9: *“Students who participate in sports tend to show more responsibility and are generally more motivated in the classroom.”* expressed their views. Under the theme of time management, P2: *“I have to organize my day carefully because of training. This helps me manage both school and sports.”* stated, while P12: *“Sometimes it becomes difficult to balance schoolwork and sports. I worry about missing lessons or falling behind.”* shared their perspective. Regarding school commitment, P3: *“Representing my school in competitions makes me feel proud and more connected to my school.”* and P10: *“Students who participate in school sports seem more attached to the school and more willing to take part in school activities.”* were expressed. Under the theme of psychosocial development, P5: *“Sports help me feel more confident and reduce my stress, especially during exam periods.”* and P16: *“My child has become more social and self-confident since starting sports.”* were stated. Concerning perceived academic risk, P13: *“I am sometimes worried that sports may cause fatigue and affect my child’s academic performance.”* expressed their concern. Finally, under the theme of adult support, P11: *“Guiding students and monitoring their progress is essential to ensure a healthy balance between academics and sports.”* and P17: *“We try to support our child in sports while also making sure that school responsibilities are not neglected.”* were stated.

The findings indicates that school sports play a multifaceted role in students’ academic, social, and personal development. The data show that participation in sports is associated with increased academic motivation, improved time management skills, and a stronger sense of school commitment. In addition, sports participation contributes to psychosocial development by enhancing self-confidence, social interaction, and stress management. However, some concerns regarding potential academic risks, particularly related to fatigue and time constraints, were also expressed, mainly by parents. Overall, the findings suggest that while school sports offer significant developmental benefits, their effectiveness is closely linked to the presence of supportive structures such as teacher guidance and parental involvement, which help students maintain a balance between academic responsibilities and sporting activities.”

Table 6 presents findings indicating that there are both shared and differing perceptions among stakeholder groups regarding the effects of school sports on academic achievement, time management, school belonging, and psychosocial development. Participant views are presented directly below in relation to the themes.

**Table 6:** Convergent and divergent perceptions across stakeholder groups.

Analytic Dimension	Convergent Perceptions	Divergent Perceptions
Sports–academics relationship	Increased motivation and discipline	Parents emphasize academic risk
School commitment	Strengthened belonging and attachment	Parents prioritize academic outcomes
Time use	Improved organization and responsibility	Students positive, parents cautious
Function of sports	Personal development	Parents stress supervision and control
Academic priority	Importance of balance	Disagreement on intensity of participation

In the context of the sport–academic relationship, participants particularly emphasized academic motivation and discipline. Students and teachers expressed this relationship through the following statements: “Being involved in sports makes me more disciplined in my lessons.” (P1), “Because of training schedules, I have to plan my day carefully.” (P2), “Students who participate in sports generally become more disciplined and responsible.” (P7), and “Students who participate in sports tend to be more motivated in the classroom.” (P9). In contrast, some parents highlighted academic risks and expressed concerns such as “I am sometimes worried that sports may lead to fatigue and affect academic performance.” (P13) and “Sometimes it is difficult to balance school and sports.” (P14).

Regarding school belonging, participants generally expressed similar and positive views. Students emphasized this sense of belonging through statements such as “Representing our school in competitions makes me feel proud.” (P3) and “I feel more connected to the school.” (P5). Likewise, teachers stated “Students who participate in school sports tend to feel more attached to the school.” (P10) and “Students’ participation in school activities increases with sports.” (P11), while parents also supported this view with the statement “Since my child started sports, they have become more interested in school.” (P16).

In the theme of time management, students noted that sports improve their planning skills. In this regard, statements such as “I learned to plan my day thanks to sports.” (P2) and “Managing both schoolwork and sports requires time management.” (P6) were prominent. Teachers similarly supported this view with “Students who do sports are able to organize their time more effectively.” (P8) and “Time management is more developed in students who participate in sports.” (P12). However, parents adopted a more cautious perspective, expressing that “It can be difficult to balance schoolwork and sports.” (P14).

In the theme of psychosocial development, the contribution of sports to personal development was highlighted. Students emphasized psychological benefits with statements such as “Sports increase my self-confidence.” (P5) and “Sports reduce my stress.” (P6). Teachers supported this perspective by stating “Sports support both the academic and personal development of students.” (P9) and “Sports contribute to students’ development in a multifaceted way.” (P11), while parents acknowledged these benefits but also emphasized control, stating “Sports should not interfere with academic responsibilities.” (P13) and “Sports have contributed to my child’s social and personal development.” (P16).

In the theme of academic priority, all stakeholder groups generally agreed on the need for balance. Students expressed this through statements such as “I try to manage both my studies and sports together.” (P1) and “It is important to keep a balance between the two.” (P2). Parents emphasized academic priority with statements such as “Academic success should always come first.” (P13), “A balance should be established between sports and schoolwork.” (P15), “It is important that our child does not neglect academic responsibilities.” (P17), and “Sports should be supported, but academic work should remain the priority.” (P18).

Overall, the findings indicate that school sports contribute in multiple ways to students’ academic, social, and personal development; however, challenges related to academic performance and time management are also acknowledged by stakeholders.

Table 7 presents representative quotations reflecting participants’ views on academic motivation, school belonging, time management, and psychosocial development. In order to directly and clearly reflect participants’ voices, their verbatim statements are presented below.

**Table 7:** Representative quotations on academic motivation and school commitment.

Theme	Stakeholder	Representative Quotation
Academic motivation	Student (S3)	“After sports practice, I feel more motivated to participate in my classes.”
School commitment	Teacher (T2)	“Students who are part of school teams feel more connected to the school.”
Psychosocial development	Student (S5)	“Sports reduce my stress and increase my self-confidence.”

In the context of academic motivation, student and teacher perspectives indicate that participation in sports enhances discipline and engagement in academic tasks. In this regard, P1 stated: “Being involved in sports helps me become more disciplined in my lessons and increases my motivation.” Similarly, P7 expressed: “Students who participate in school sports are generally more disciplined and more motivated toward their lessons.”

Regarding school belonging, participants emphasized that sports strengthen students’ connection to their school. P3 noted: “Representing our school in competitions makes me feel proud and increases my sense of belonging to the school.” Likewise, P10 stated: “Students who are part of school teams develop a stronger sense of belonging to the school.”

In terms of time management, students and teachers highlighted that sports participation contributes to planning and organization skills. P2 explained: “Because of training sessions, I have to plan my day, which helps me manage my time better.” Supporting this, P8 stated: “Students who engage in sports are able to use their time in a more planned and organized way.”

With respect to psychosocial development, participants emphasized the positive effects of sports on stress management and self-confidence. P5 stated: “Sports reduce my stress and increase my self-confidence.” From a parental perspective, P16 expressed: “Since my child started sports, they have become more social and more self-confident.”

Concerning perceived academic risks, parents particularly drew attention to potential negative effects of sports participation. P13 noted: “I think sports can sometimes cause fatigue and negatively affect academic performance.”

Finally, in the theme of academic prioritization, parents emphasized the importance of maintaining a balance between sports and academic responsibilities. P17 stated: “We ensure that my child both participates in sports and does not neglect academic responsibilities.”

Overall, these quotations demonstrate that while students and teachers primarily emphasize the motivational and organizational benefits of sports participation, parents tend to focus more on maintaining balance and addressing potential academic risks. Beyond the descriptive presentation of themes, a comparative interpretation of stakeholder perspectives reveals both convergences and divergences across groups. Students primarily emphasize personal motivation and social aspects of school sports, teachers highlight academic engagement and behavioral outcomes, and parents focus on developmental and supportive dimensions. These differences suggest that each stakeholder group approaches school sports from distinct but complementary perspectives. Overall, the findings indicate that school sports function as a multidimensional phenomenon that is perceived differently depending on stakeholders’ roles and experiences, yet these perspectives collectively contribute to a more holistic understanding of its impact.

Table 8 presents representative quotations reflecting participants’ views on time management, perceived academic risks, and the need for balance between sports and academic responsibilities. In order to directly reflect participants’ voices, their verbatim statements are presented below.

**Table 8:** Representative quotations on time management and perceived academic risks.

Theme	Stakeholder	Representative Quotation
Time management	Student (S1)	“Training taught me how to plan my time better.”
Perceived academic risk	Parent (P4)	“On days with intensive training, lessons may sometimes be neglected.”
Need for balance	Teacher (T5)	“When the balance between sports and academics is managed well, problems do not arise.”

In relation to time management, the student perspective highlights the role of sports participation in developing planning skills. In this context, P1 stated: “Training taught me how to plan my time better.” This statement indicates that involvement in sports contributes to students’ ability to organize their daily routines and manage competing demands.

Regarding perceived academic risks, the parental perspective emphasizes potential negative effects of intensive training on academic engagement. P4 expressed this concern by stating: “On days with intensive training, lessons may sometimes be neglected.” This reflects parents’ sensitivity to the possibility that sports participation may interfere with academic responsibilities under certain conditions.

In terms of the need for balance, the teacher perspective underscores the importance of maintaining equilibrium between academic and sporting activities. P5 noted: “When the balance between sports and academics is managed well, problems do not arise.” This statement suggests that effective coordination between these two domains can mitigate potential challenges and support student development.

Overall, the quotations in Table 7 illustrate that while students primarily emphasize the developmental benefits of sports for time management, parents tend to highlight potential academic risks, and teachers stress the importance of maintaining a balanced approach between sports and academics.

## Discussion

The findings derived from Table 1 indicate that school sports are perceived by students, teachers, and parents as a multifaceted context influencing both academic achievement and school commitment. This multidimensional perception can be partly explained by the differing roles and expectations of each stakeholder within the school ecosystem; while students primarily experience school sports as a source of motivation and social belonging, parents tend to evaluate it through the lens of academic risk and time allocation, and teachers interpret it in relation to instructional balance and student engagement, consistent with prior research on stakeholder-based differences in educational perceptions [35,36]. These differing perspectives may also reflect broader cultural and institutional priorities in the Turkish educational context, where high-stakes examinations and strong academic achievement orientation often shape parental concerns regarding time spent on non-academic activities [37,38]. From a theoretical perspective, these dynamics can be interpreted through ecological systems theory, which emphasizes the interaction of individual, relational, and contextual factors in shaping student development [39]. These findings align with and extend existing literature by illustrating how academic and motivational outcomes associated with sports participation are shaped through interrelated personal, relational, and contextual processes rather than through participation alone, suggesting that the impact of school sports emerges through a socially constructed and interactional framework rather than a purely individual-level effect.

One of the most salient findings concerns the perceived enhancement of academic motivation among students involved in school sports. Across stakeholder groups, sports participation was associated with increased focus, goal orientation, and engagement in learning. This finding can be explained through the mechanism that structured and goal-oriented sport environments foster self-regulation skills such as discipline, persistence, and time management, which may subsequently transfer to academic settings, consistent with self-regulated learning and life skills transfer perspectives in sport psychology literature [40]. This finding is also in line with school commitment and engagement theory, which emphasizes that participation in meaningful school-related activities can strengthen students’ emotional and cognitive investment in academic tasks [8]. From this perspective, school sports appear to function as a motivational resource that reinforces students’ willingness to exert effort and persist in academic challenges by supporting basic psychological needs such as competence and relatedness, as proposed in Self-Determination Theory [41]. Previous studies have similarly reported that students who participate in organized sports demonstrate higher academic motivation and engagement compared to non-participants [4,9], suggesting that this relationship is relatively stable across different educational contexts and student populations.

Time management emerged as a more complex and ambivalent theme, revealing important differences across stakeholder perspectives. While students frequently framed sports participation as a mechanism for developing planning skills and self-discipline, parents were more inclined to emphasize the potential difficulty of balancing academic demands with training and competition schedules. This difference may stem from varying levels of exposure to students’ daily routines and differing role expectations, and is also consistent with developmental literature highlighting role conflict between family and individual expectations during adolescence [42,43]. This divergence reflects earlier findings suggesting that sports participation can simultaneously foster self-regulatory skills while also introducing competing demands on students’ time and energy [5,6]. From a developmental perspective, these dual outcomes can be explained through the tension between skill acquisition and role overload during adolescence, a period characterized by increased responsibilities that may both support and challenge self-management capacities [44]. Importantly, the present findings suggest that these outcomes are not inherent to sports participation itself but are contingent on contextual factors such as scheduling, academic support, and adult supervision. In this sense, the role of teachers and coaches as coordinating agents becomes critical in reducing time-related conflicts through planning, feedback, and adaptive scheduling practices, consistent with supportive coaching approaches in sport pedagogy [45]. This interpretation aligns with ecological approaches to school engagement, which highlight the role of environmental supports in shaping students’ academic behaviors [10].

School commitment appeared as a key lens through which the impact of school sports was understood, particularly in the narratives of students and teachers. Rather than being expressed in abstract terms, this sense of commitment was grounded in concrete experiences such as representing the school in competitions, belonging to a team, and receiving recognition within the school environment. These experiences seem to transform sports participation into a socially meaningful space where students negotiate their identity within the school community, a process that has been discussed within social identity and school belonging literature [46,47]. In this sense, commitment is not only an emotional outcome but also a relational process shaped through interaction with peers, teachers, and the wider school culture. These findings resonate with the emotional dimension of school commitment as conceptualized by Fredricks et al. [8], which emphasizes feelings of attachment, acceptance, and identification with the school community. Prior research has similarly shown that participation in extracurricular sports can strengthen students’ sense of belonging and reduce disengagement by offering structured opportunities for recognition and social integration within the school setting [1,5]. At the same time, the prominence of school representation in the findings suggests that institutional visibility and collective achievement may play a particularly salient role in reinforcing students’ connection to school in this context, consistent with research emphasizing the role of recognition and status in adolescent school engagement [1].

Beyond academic and motivational outcomes, the findings also point to the role of school sports in shaping students’ psychosocial development, particularly in areas such as self-confidence, interpersonal competence, and stress regulation. Rather than emerging automatically from participation, these gains appear to be cultivated through the quality of interactions and the structure of the sporting experience within the school setting. In particular, supportive feedback, opportunities for meaningful participation, and the presence of constructive peer dynamics seem to function as key conditions through which these psychosocial benefits are realized, aligning with research on the developmental climate of youth sport environments [3]. These observations are broadly consistent with positive youth development perspectives, which frame sport as a context for fostering competence, confidence, and social connectedness [2,3], yet the current findings suggest a more context-sensitive interpretation. Specifically, the extent to which such developmental outcomes are achieved appears to depend on how sports activities are organized, integrated into the school culture, and guided by teachers and coaches. In this regard, adult support does not merely accompany participation but actively shapes the developmental value of sport by structuring experiences, modelling social behaviors, and regulating stress-related demands, as also emphasized in studies on coach-created motivational climates [48].

A particularly significant contribution of the present study lies in foregrounding adult support not merely as a facilitating condition but as an active structuring force that shapes how school sports are experienced and interpreted. The accounts of teachers and parents suggest that this influence operates through distinct yet complementary pathways: teachers tend to engage in ongoing regulation of students’ dual roles by monitoring academic progress, adjusting expectations, and coordinating schedules, whereas parents contribute by framing sports participation within broader value systems related to responsibility, balance, and long-term educational priorities. In this sense, school commitment emerges less as an individual disposition and more as a relationally produced outcome, shaped through sustained patterns of guidance, communication, and negotiated expectations among students, teachers, and families. This interpretation aligns with prior work that conceptualizes engagement as co-constructed through such interactions [4,10], but the present findings extend this view by illustrating the specific mechanisms through which adults translate institutional demands into manageable and meaningful experiences for students. In particular, practices such as feedback, expectation alignment, and flexible coordination appear to reduce potential tensions between academic and athletic roles, thereby enabling students to maintain continuity in both domains. This perspective is also consistent with research on parental involvement and teacher support, which highlights that students’ academic engagement is strengthened when adults provide structured guidance while preserving autonomy [49,50].

The findings derived from Table 2 suggest that the perceived effects of school sports on academic achievement and school commitment are not only shaped by shared understandings but also differentiated through stakeholder-specific interpretive frames. While students, teachers, and parents converge in associating sports participation with increased academic motivation and discipline, this agreement appears to rest on different underlying rationales tied to their roles within the educational process. For students, these gains are often experienced through the routine demands and immediate feedback embedded in sport participation; for teachers, they are observed in terms of behavioral consistency and classroom engagement; whereas parents tend to interpret them in relation to responsibility and long-term academic orientation. This layered convergence helps explain why similar outcomes are recognized across groups, yet justified through distinct perspectives. Such patterns are consistent with prior research indicating that structured extracurricular activities foster responsibility, goal orientation, and self-regulatory behaviors in adolescents [43,51]. However, the present findings move beyond a generalized account by suggesting that the transfer of these competencies to academic contexts is not merely a by-product of participation, but is mediated through processes of internalization shaped by repeated practice, social expectations, and evaluative feedback, as emphasized in social-cognitive perspectives on behavioral regulation [52]. In this sense, organized sports can be understood as structured micro-contexts where norms, routines, and performance standards are continuously reinforced, enabling students to adapt these patterns to academic tasks under conditions where expectations remain consistent and meaningful.

The shared emphasis on enhanced school belonging further supports the view that school sports operate as a form of social integration that extends beyond participation itself. However, the findings suggest that this sense of belonging is not produced in a uniform way; rather, it emerges through repeated interactions in which students are recognized, assigned roles, and publicly positioned within the school community, a process consistent with social identity theory which emphasizes the role of group membership in shaping self-concept [46,53]. In this respect, belonging appears to be socially constructed through everyday school practices rather than simply experienced as an internal feeling. Research on school connectedness similarly indicates that participation in school-based activities strengthens students’ emotional ties to school and contributes to more positive school attitudes [54,55]. Yet, the present findings extend this view by suggesting that connectedness is shaped not only by participation but also by the quality of social recognition and the visibility students gain through representing their school in structured settings such as competitions, aligning with literature highlighting the importance of status and recognition in adolescent school engagement [1].

Despite these areas of alignment, the divergent perceptions identified among stakeholders offer a particularly revealing dimension of the findings. Parents’ heightened concern regarding academic strain, fatigue, and time pressure is not merely an expression of caution, but appears to reflect a broader interpretive framework shaped by culturally and institutionally embedded expectations that prioritize academic success within competitive examination-oriented schooling systems. In this respect, parental evaluations seem to be informed by both immediate observations of students’ workload and broader societal narratives about educational attainment and social mobility. This aligns with research highlighting that intensive extracurricular involvement may be perceived as a potential cost when academic demands are high, particularly in contexts characterized by strong performance pressures and limited recovery time [56,57]. From a developmental standpoint, these concerns can also be understood as part of parents’ protective orientation during adolescence, where balancing opportunity enhancement with risk avoidance becomes a central evaluative stance [58].

In contrast, students’ more positive interpretations of time management and balance reflect a distinct experiential positioning that frames sports participation not as a competing demand but as a structured context for negotiating responsibility and autonomy. This perspective resonates with studies suggesting that adolescents often perceive extracurricular involvement as an opportunity to develop organizational skills, self-regulation, and a sense of agency in managing multiple roles [59]. However, the present findings suggest that this perceived benefit is closely linked to the immediacy of lived experience: students tend to evaluate time demands through day-to-day engagement, where skill acquisition is reinforced through repetition and direct feedback, rather than through anticipatory concerns about long-term academic strain. From this standpoint, sports participation may enhance rather than undermine academic functioning by fostering self-discipline and a sense of control over competing demands. This divergence highlights that interpretations of academic impact are not merely attitudinal differences, but are shaped by developmental positioning, role expectations, and the extent of direct involvement in managing time-related pressures, consistent with developmental theories emphasizing age- and role-based differences in perception and self-regulation [44].

Teachers’ mediating stance plays a particularly salient analytical role in understanding how school sports intersect with academic engagement. Rather than adopting a static role, teachers appear to engage in a continuous balancing process shaped by their ongoing observational judgments of students’ academic engagement, fatigue levels, and classroom responsiveness, reflecting research on teacher cognition and in-the-moment pedagogical decision-making [60]. This positioning enables teachers to translate institutional expectations into practical adjustments, often through workload calibration, feedback provision, and informal coordination between academic and athletic demands. In this sense, mediation is embedded in everyday school routines and reflects the adaptive nature of teaching practice in complex school environments. Previous studies similarly suggest that educators function as key intermediaries in balancing academic and extracurricular expectations through monitoring, feedback, and informal regulation of students’ commitments [61,62]. However, the present findings extend this view by indicating that such mediation is situational rather than fixed, varying according to perceived changes in students’ readiness, performance, and well-being, which aligns with literature on adaptive teaching and contingent instructional decision-making [63]. This reinforces the idea that teacher guidance constitutes a critical contextual factor in determining whether sports participation enhances academic engagement or becomes a competing demand.

Differences in time use and academic prioritization further point to the central role of family-based belief systems in shaping how school sports are evaluated. Rather than reflecting individual parental caution alone, these attitudes appear to be embedded in broader parental involvement processes through which expectations, values, and perceived responsibilities are transmitted to adolescents. In this sense, parents interpret sports participation through a framework in which academic achievement is closely tied to future social mobility, making time allocation a sensitive domain of decision-making. This aligns with research emphasizing that parental beliefs about education and success significantly shape the degree and manner in which extracurricular activities are supported or constrained [64,65].

While stakeholders consistently identify time pressure, academic workload, physical fatigue, and difficulties in maintaining balance between athletic and academic responsibilities, these concerns appear to reflect more than isolated stressors; they point to the broader organizational conditions of schooling in which multiple performance domains operate simultaneously and often compete for limited cognitive and temporal resources. From this perspective, strain emerges not simply from participation in sports, but from the interaction between training schedules, assessment cycles, and expectations for continuous academic performance. These findings reinforce the view that the educational impact of school sports is contingent upon the conditions under which participation is organized and supported [6], while also aligning with research on role strain and competing demands in adolescent developmental contexts [9]. Accordingly, the outcomes of sports participation should be understood as dynamically dependent on how effectively schools and families coordinate these overlapping responsibilities rather than as inherent consequences of involvement itself.

Students’ accounts of reduced study time during examination periods and difficulties in managing academic responsibilities should be understood not merely as scheduling conflicts but as indicators of competing developmental and institutional demands that characterize adolescent school life. In contexts where high-stakes examinations structure academic progression, time becomes a constrained resource that is simultaneously allocated to performance evaluation and skill development in sports, creating periods of intensified role overlap. Such overlap can increase cognitive load and emotional strain, particularly when external regulatory supports (e.g., coordinated scheduling or academic guidance systems) are limited, thereby affecting learning efficiency and sustained attention [9]. However, the students’ consistent emphasis on personal planning, prioritization, and self-monitoring strategies suggests that these pressures are not experienced uniformly; rather, they are mediated by individual differences in self-regulatory capacity. This aligns with theoretical models of self-regulated learning, which conceptualize academic success as dependent on learners’ ability to set goals, monitor progress, and adapt strategies across changing contextual demands [52].

Teachers primarily interpret academic risks through directly observable classroom indicators, such as fatigue-related concentration difficulties, fluctuations in participation, reduced task persistence, and episodic absenteeism during intensive competition periods. This reliance on visible behavioral cues reflects the situated and experiential nature of teacher cognition, in which professional judgments are constructed through continuous classroom observation and interpretation of student behavior [60,66]. Such perceptions are consistent with physiological and psychological evidence indicating that excessive physical load combined with insufficient recovery can impair attentional control, working memory efficiency, and sustained cognitive performance [67]. However, teachers’ accounts do not position these difficulties as inherent consequences of sports participation itself; instead, they attribute them to gaps in institutional coordination across administrative planning, coaching schedules, and academic expectations. From an organizational perspective, this aligns with ecological views of schooling, which emphasize that student outcomes are shaped by the degree of coherence and alignment across interacting school systems rather than isolated practices [68]. In this regard, the findings suggest that the educational impact of school sports is shaped less by participation per se and more by the organizational integration of school actors, highlighting institutional coordination as a decisive factor in whether sports function as a supportive structure or a disruptive demand in students’ academic trajectories.

Parents expressed a more cautious and future-oriented stance, emphasizing concerns that sports participation might overshadow academic priorities, particularly within examination-driven education systems where educational success is closely linked to high-stakes selection processes. This orientation appears to reflect not only individual concern but also a culturally embedded logic in which academic performance functions as a form of long-term social and economic capital, shaping parental decision-making regarding time allocation and extracurricular involvement. Within this framework, sports participation is evaluated through a preventive lens in which potential academic opportunity costs are continuously assessed against perceived future educational trajectories. This perspective aligns with sociological research indicating that parental evaluations of extracurricular activities are strongly influenced by concerns about academic capital formation and future mobility pathways [69], as well as broader studies on cultural capital and educational reproduction [70]. Importantly, parents did not reject sports participation; rather, they advocated for structured, supervised, and balanced engagement, highlighting the need for ongoing academic monitoring and regulated involvement to ensure alignment between athletic activities and educational expectations.

Students interpret risks primarily through immediate academic workload and short-term performance demands, teachers construct their judgments through instructional flow, classroom regulation, and broader school organizational coordination processes, reflecting research on instructional leadership and the organizational role of teachers in mediating student learning environments [71]. Parents, in contrast, evaluate potential risks through a future-oriented lens emphasizing long-term academic trajectories and educational mobility, aligning with developmental and sociological accounts of role-based perception differences during adolescence [1]. These differentiated perspectives suggest that “risk” is not a fixed attribute of school sports participation but a socially constructed interpretation shaped through role-based engagement with schooling. This pattern supports ecological perspectives on educational engagement, which emphasize that student outcomes are produced through dynamic interactions across relational, institutional, and contextual systems rather than isolated individual experiences [68,72]. From this standpoint, school sports function as a multi-layered setting in which meaning is continuously negotiated across stakeholders taking on different role within the same educational environment.

## Conclusion

This qualitative descriptive study examined the perceived effects of school sports on students’ academic achievement and school commitment through the perspectives of students, teachers, and parents. By adopting a multi-informant focus group design, the study moved beyond single-source accounts and provided a more nuanced understanding of how school sports are experienced and interpreted within everyday school contexts.

Overall, the findings indicate that school sports are widely perceived as contributing positively to students’ academic motivation, sense of discipline, and commitment to school. Across stakeholder groups, participation in school sports was associated with increased engagement in learning, stronger identification with the school, and enhanced psychosocial competencies such as self-confidence, social interaction skills, and stress management. These outcomes were most evident when sports participation was embedded within a supportive school environment characterized by guidance, monitoring, and constructive adult involvement.

At the same time, the study highlights that the educational value of school sports is not unconditional. Students, teachers, and parents identified potential challenges related to time pressure, physical fatigue, and difficulties in balancing academic and athletic demands, particularly during periods of intensive training and competition. Importantly, these challenges were not perceived as inherent to sports participation itself, but rather as consequences of insufficient coordination between academic and athletic structures. This finding underscores the central role of institutional organization and inter-stakeholder communication in shaping the academic implications of school sports.

The comparative analysis across stakeholder groups revealed meaningful differences in how benefits and risks were interpreted. Students tended to emphasize personal experiences and self-regulatory strategies, teachers focused on classroom engagement and instructional processes, and parents evaluated sports participation through the lens of long-term academic trajectories. These differentiated perspectives demonstrate that school commitment is a relational and contextual phenomenon, co-constructed through interactions among students, families, educators, and school practices. The added value of the multi-informant design lies precisely in illuminating these points of convergence and divergence, which would remain obscured in single-perspective studies.

From a theoretical standpoint, the findings support multidimensional models of school commitment by illustrating how emotional, behavioral, and cognitive dimensions are simultaneously influenced by sports participation. School sports appear to function as a meaningful context through which students negotiate belonging, responsibility, and academic engagement, provided that appropriate structural supports are in place. Rather than positioning sports and academics as competing domains, the results suggest that their relationship is dynamic and highly dependent on contextual conditions.

In practical terms, the findings point to the need for school-level policies that integrate sports programs with academic priorities. Effective scheduling, collaboration among teachers, coaches, and administrators, and transparent communication with families are essential to maximizing benefits and minimizing risks. Supporting students’ self-regulation skills and providing guidance during academically demanding periods may further enhance the compatibility of sports participation with academic success.

While the study offers valuable insights, its findings should be interpreted in light of certain limitations, including the single-school context and reliance on perceived rather than objective academic indicators. Future research could build on these findings by incorporating longitudinal designs, multiple school settings, and mixed-method approaches to further explore how school sports contribute to academic and motivational outcomes across diverse contexts.

In conclusion, this study demonstrates that school sports hold significant potential to support students’ academic achievement and school commitment, not as an automatic outcome of participation, but as a contextually mediated process shaped by relational, organizational, and individual factors. Recognizing and addressing these conditions is essential for realizing the educational value of school sports in contemporary school systems.

Finally, although the study provides valuable findings, it has certain limitations due to being conducted within a single-school context and relying on perceptions rather than objective academic indicators. Therefore, the findings should not be interpreted as generalizable universal results but rather as qualitative insights produced within a specific context.

### Recommendations

Based on the findings of this study, the following recommendations are proposed to enhance the positive contributions of school sports to students’ academic achievement and school commitment, while minimizing potential challenges:

**1. Recommendations for educational policy**

- School sports should be positioned as a complementary component of academic programs rather than as an activity competing with academic priorities. Educational policies should promote integrated planning that aligns sports schedules with academic calendars.
- Policy frameworks should acknowledge the multidimensional contributions of school sports, including their role in fostering academic motivation, self-regulation, and school commitment, alongside physical development.

**2. Recommendations for school administration and institutional practices**

- School administrators should establish systematic coordination mechanisms among teachers, coaches, counselors, and administrators to support students’ academic–athletic balance.
- Flexible academic arrangements may be implemented during periods of intensive sports participation (e.g., competitions or tournaments) to reduce time pressure and academic strain.
- chools may develop guidance-based monitoring systems to identify students at risk of imbalance and to provide timely academic or psychosocial support.

**3. Recommendations for teachers and coaches**

- Physical education teachers and coaches should design training programs that take students’ academic responsibilities into account and avoid excessive workloads during critical academic periods.
- Subject teachers should adopt instructional and assessment strategies that support student-athletes’ continued engagement with academic tasks.
- Educators are encouraged to explicitly connect competencies developed through sports participation—such as discipline, persistence, and goal orientation—to academic learning processes.

**4. Recommendations for parents and families**

- Parents should be encouraged to view school sports not as a threat to academic success but as a potential resource that, when appropriately managed, can support students’ overall development.
- Families should promote balanced expectations and support students in developing effective time management and self-regulation skills.
- Strengthening school–family communication is essential to ensure shared understanding of students’ academic and athletic commitments.

**5. Recommendations for students**

- Students should be supported in developing self-regulatory skills, including time management, planning, and goal setting, to effectively balance academic and sports-related demands.
- Schools should foster students’ awareness of sports as a context for personal growth that can positively contribute to academic engagement and school commitment.

**6. Recommendations for future research**

- Future studies should examine the effects of school sports across different school types, age groups, and socio-cultural contexts to enhance the transferability of findings.
- Quantitative and mixed-methods research designs are recommended to complement qualitative insights with objective indicators of academic achievement.
- Longitudinal studies may provide deeper understanding of the long-term academic and psychosocial impacts of sustained participation in school sports.

### Strengths

This study has several notable strengths that enhance its methodological rigor and theoretical contribution. First, the use of a multi-informant qualitative design constitutes a major strength. By incorporating the perspectives of students, teachers, and parents, the study moves beyond single-informant approaches and provides a more comprehensive understanding of how school sports are perceived to influence academic achievement and school commitment. This comparative perspective allows for the identification of both convergent and divergent interpretations, thereby capturing the relational and contextual nature of school sports within the educational environment.

Second, the study demonstrates conceptual clarity by explicitly grounding the analysis in school commitment theory. Rather than treating engagement, belonging, and attachment as interchangeable constructs, the study adopts school commitment as the primary analytical framework and interprets findings across emotional, behavioral, and cognitive dimensions. This theoretically anchored approach strengthens the coherence between the research aims, data analysis, and interpretation of findings.

Third, the qualitative descriptive design, supported by focus group interviews and systematic descriptive and inductive content analysis, represents a methodological strength. This approach is well suited to capturing participants’ experiences and viewpoints in a clear and accessible manner, while still allowing for analytic depth. The use of direct quotations enhances transparency and supports the credibility of the findings by grounding interpretations firmly in participants’ own accounts. To enhance the transparency of the coding process, an additional step of independent coding was conducted. A subset of the transcripts was independently coded by a second researcher, and the coding results were compared with those of the first author. Discrepancies were discussed and resolved through consensus, ensuring consistency and reliability in the coding scheme. No qualitative data analysis software was used; instead, coding and categorization were conducted manually to allow for close engagement with the data. The development of themes followed a systematic process from codes to categories and then to higher-order themes. Initially, similar codes were grouped into categories based on shared meanings. These categories were then further abstracted into broader themes by examining conceptual relationships and patterns across the dataset.

Another important strength lies in the study’s contextual contribution. Conducted within a Turkish high school context, the research addresses a setting that remains underrepresented in the international literature on school sports and academic outcomes. By situating the findings within an examination-oriented education system, the study offers context-sensitive insights that extend existing theoretical discussions and highlight how cultural and institutional conditions shape the perceived role of school sports.

Finally, the study’s focus on perceived academic achievement, rather than solely on objective performance indicators, provides a nuanced understanding of how academic outcomes are experienced and interpreted by different stakeholders. This emphasis contributes to a deeper appreciation of motivational, relational, and organizational processes that mediate the relationship between school sports and educational engagement.

### Limitations

Despite its methodological rigor and conceptual grounding, this study has several limitations that should be acknowledged when interpreting the findings.

First, the study is based on a qualitative descriptive design with a relatively small and context-specific sample. Data were collected from students, teachers, and parents within a single high school context in Sakarya, Türkiye. While this design enabled an in-depth exploration of stakeholder perceptions, the findings are not intended to be statistically generalizable. Instead, they provide context-bound insights that may be transferable to similar educational settings. Readers should therefore exercise caution when applying the results to different school systems or cultural contexts.

Second, academic achievement was examined as a perceived construct rather than through objective indicators such as grades, standardized test scores, or academic records. Although this approach is consistent with the qualitative aims of the study and allows for an exploration of meaning-making processes, it limits the ability to directly link school sports participation to measurable academic outcomes. Future research could benefit from integrating qualitative perceptions with quantitative academic data to strengthen explanatory depth.

Third, data were collected through focus group interviews, which may have influenced participants’ responses due to group dynamics. In particular, students may have been affected by peer presence, and parents or teachers may have moderated their statements to align with socially acceptable views. Although efforts were made to encourage open discussion, the possibility of conformity or social desirability bias cannot be fully ruled out.

Fourth, while the study employed a multi-informant design, it did not include perspectives from school administrators or coaches who are directly involved in organizing school sports programs and scheduling academic–athletic demands. Their inclusion could have provided additional insights into institutional decision-making processes and coordination mechanisms that shape students’ experiences.

Finally, the cross-sectional nature of the study limits insight into how perceptions of school sports and their academic implications may change over time. Longitudinal qualitative or mixed-methods studies could offer a more dynamic understanding of how school commitment and perceived academic outcomes evolve with sustained sports participation.

## Supporting information

S1 File Anonymized dataset. (RAR)
