# Supplementary material for: Perceived academic achievement and school commitment in the context of school sports: A qualitative study based on students’, teachers’, and parents’ perspectives
Source: PLoS One. 2026 Jun 29;21(6):e0351877. doi: 10.1371/journal.pone.0351877 (PMC13313331; doi:10.1371/journal.pone.0351877)
Supplement: Attachment — Submitted filename: Response to Reviewers.docx [file pone.0351877.s002.docx]

1. **Keywords**: In line with your recommendation, the keywords have been revised to avoid repetition of terms already included in the title and to enhance the discoverability of the article.
2. **Introduction**: In response to the comment regarding repetition, the Introduction section has been carefully revised to reduce redundancy, particularly in the parts related to the definition of school commitment and the role of sports. Overlapping statements were consolidated and streamlined to improve clarity and coherence.
3. **Ethics**: We have included a comprehensive ethics statement in the Methods section of the revised manuscript. The statement now clearly specifies the full name of the institutional ethics committee that approved the study (Sakarya University of Applied Sciences Ethics Committee), along with the approval date (15.11.2024) and reference number (E-26428519-050.99-149279 / Decision 50/22). In addition, we have clarified that informed consent was obtained from all participants prior to data collection.
4. As noted by the esteemed reviewer, the need to properly support the statements with appropriate references and to clarify the attribution of the claims has been taken into consideration. Accordingly, the relevant section of the manuscript has been revised, and the statements on page 6 (not page 12) have been strengthened by incorporating appropriate references from the literature regarding the perceived effects of school sports on students, teachers, and parents.
5. Regarding Table 1, the term “Athleticism” has been revised for clarity. It has been corrected to “Athletics” to more accurately reflect the sport category intended in the manuscript. Regarding Table 3, the column labeled “Branch” refers to the professional field or area of expertise of the parents (e.g., Mathematics, History, Physics, etc.). To avoid any ambiguity, this term has been clarified in the revised manuscript, and the label has been updated to more clearly indicate “Profession” instead of “Branch.”
6. **The focus group interview questions**: In line with the reviewer’s recommendation, the manuscript has been substantially revised. The focus group interview questions have been removed from the main text. Instead, greater emphasis has been placed on presenting participants’ voices directly within the findings section. Participants’ statements are now presented as verbatim quotations without summarization or paraphrasing, and these direct quotes are provided under the relevant tables.
7. In addition, in line with the reviewer’s suggestion concerning epistemological positioning, we have clarified the methodological stance of the study. The revised manuscript explicitly frames the study within a descriptive qualitative research design. While participants’ experiences and perspectives are explored in depth, the study does not adopt a phenomenological framework; rather, it aims to provide a structured and contextually grounded description of these perspectives.
8. **The Sampling** section of the manuscript has been revised to include detailed information on how participants were identified, approached, and recruited. Specifically, it has been clarified that participants were identified through collaboration with the school administration and physical education teachers, and then invited to participate on a voluntary basis. Furthermore, it has been explicitly stated that all eligible participants (n = 18) were invited to the study and all agreed to participate. In addition, the revised version now clearly reports that no participants declined participation or withdrew from the study, and that informed consent was obtained from all participants prior to data collection.
9. We agree that data saturation is a critical aspect of qualitative research and should be explicitly addressed to justify sample size. In response to this comment, we have revised the manuscript to include a clear explanation regarding data saturation. Specifically, we now state that data collection continued until thematic saturation was reached, meaning that no new themes or meaningful insights emerged from the interviews. The sample size of six participants per stakeholder group was considered sufficient, as participants provided consistent and repetitive patterns in their responses. This justification has been added to the revised manuscript, and it aligns with qualitative research principles, where sample size is determined by information richness rather than statistical representativeness.
10. An additional explanation has been incorporated into the manuscript to justify why a single focus group was conducted for each stakeholder group. In qualitative research, the number of focus groups is determined by the principle of data saturation and the depth of information obtained rather than numerical representativeness. In the present study, each stakeholder group (students, teachers, and parents) was relatively homogeneous in nature, and participants provided consistent and repetitive patterns of responses during the discussions. Therefore, a single focus group per stakeholder category was considered sufficient to capture a range of perspectives while still allowing in-depth interaction among participants. Furthermore, methodological literature indicates that small and well-structured focus groups, when properly moderated, can yield rich and meaningful qualitative data, particularly when participants share similar contextual characteristics.
11. We have revised the manuscript to include illustrative examples of both main interview questions and follow-up probing prompts. Additionally, we clarified how the interview questions were refined during the pilot study to improve clarity and accessibility.
12. **Coding prosedure**: The manuscript has been revised to provide a more detailed description of the coding procedure. Specifically, we clarified that a subset of the data was independently coded by a second researcher, and discrepancies were resolved through discussion and consensus. Although inter-coder agreement was not calculated quantitatively, consistency was ensured through this iterative comparison process. Additionally, we expanded the explanation of how codes were grouped into categories and subsequently abstracted into higher-order themes, providing a clearer account of the inductive analysis process. To further enhance transparency, a coding tree (Table 4) has been included to illustrate the progression from raw data to codes, categories, and themes.
13. **The Findings** section has been revised to include a more explicit comparative interpretation of stakeholder perspectives. An additional paragraph was added to highlight both convergences and divergences among students’, teachers’, and parents’ views, moving beyond a purely descriptive presentation and strengthening the interpretive depth of the analysis.
14. Coding tree (coding hierarchy) has been added to the manuscript to enhance the transparency and traceability of the qualitative data analysis process.

**DISCUSSION**

In response to the reviewer’s concerns, several substantial revisions have been made.

First, the Discussion section has been revised to move beyond a descriptive summary of findings and to provide a more interpretive and theoretically grounded analysis. Specifically, additional explanation has been included to clarify the mechanisms underlying divergent perceptions among students, teachers, and parents. These differences are now more explicitly discussed in relation to cultural (e.g., examination-oriented education system), institutional (e.g., school organization and coordination), and developmental (e.g., adolescent role perception) factors.

Second, the mediating role of teachers has been strengthened by specifying concrete mechanisms such as classroom monitoring, feedback processes, scheduling coordination, and adaptive instructional judgment. This revision provides a clearer understanding of how teachers function as key intermediaries between academic and athletic demands.

Third, the contextual limitations of the study have been expanded in both the Discussion and Conclusion sections. In line with the reviewer’s suggestion, we have explicitly emphasized that the findings are context-specific, exploratory in nature, and not intended to be generalizable beyond the single-school setting in which the study was conducted. This clarification has been integrated to avoid overgeneralization and to better align the interpretation of results with the qualitative design of the study.

Fourth, the contribution of the multi-stakeholder design has been further articulated. The revised manuscript now more clearly highlights that the inclusion of students, teachers, and parents enables the identification of both convergent and divergent interpretations of school sports, offering insights that would not be visible in single-perspective studies.

Finally, the Conclusion section has been revised to ensure that the interpretive claims are appropriately bounded by the exploratory nature of the research and its contextual limitations.

*Conclusion section has been revised to more clearly emphasize the exploratory nature of the study and its context-specific character. The revised version explicitly states that the findings are based on a single-school setting and should not be interpreted as generalizable results.
