# Supplementary material for: Perceived academic achievement and school commitment in the context of school sports: A qualitative study based on students’, teachers’, and parents’ perspectives
Source: PLoS One. 2026 Jun 29;21(6):e0351877. doi: 10.1371/journal.pone.0351877 (PMC13313331; doi:10.1371/journal.pone.0351877)
Supplement: Peer review history

# Peer review: Decision Letter 0

**DOI:** 10.1371/journal.pone.0351877.r001

**Authors:** Francesca D'Elia

31 Mar 2026

PONE-D-26-01877

Perceived Academic Achievement and School Commitment in the Context of School Sports: A Qualitative Study Based on Students’, Teachers’, and Parents’ Perspectives

PLOS One

Dear Dr. Taş,

Thank you for submitting your manuscript to PLOS ONE. After careful consideration, we feel that it has merit but does not fully meet PLOS ONE’s publication criteria as it currently stands. Therefore, we invite you to submit a revised version of the manuscript that addresses the points raised during the review process.

Please submit your revised manuscript by May 15 2026 11:59PM**.**  If you will need more time than this to complete your revisions, please reply to this message or contact the journal office at plosone@plos.org. When you're ready to submit your revision, log on to https://www.editorialmanager.com/pone/ and select the 'Submissions Needing Revision' folder to locate your manuscript file.

Please include the following items when submitting your revised manuscript:

- A letter that responds to each point raised by the academic editor and reviewer(s). You should upload this letter as a separate file labeled 'Response to Reviewers'.
- A marked-up copy of your manuscript that highlights changes made to the original version. You should upload this as a separate file labeled 'Revised Manuscript with Track Changes'.
- An unmarked version of your revised paper without tracked changes. You should upload this as a separate file labeled 'Manuscript'.

If you would like to make changes to your financial disclosure, please include your updated statement in your cover letter. Guidelines for resubmitting your figure files are available below the reviewer comments at the end of this letter.

If applicable, we recommend that you deposit your laboratory protocols in protocols.io to enhance the reproducibility of your results. Protocols.io assigns your protocol its own identifier (DOI) so that it can be cited independently in the future. For instructions see: https://journals.plos.org/plosone/s/submission-guidelines#loc-laboratory-protocols. Additionally, PLOS ONE offers an option for publishing peer-reviewed Lab Protocol articles, which describe protocols hosted on protocols.io. Read more information on sharing protocols at https://plos.org/protocols?utm_medium=editorial-email&utm_source=authorletters&utm_campaign=protocols.

As the corresponding author, your ORCID iD is verified in the submission system and will appear in the published article. PLOS supports the use of ORCID, and we encourage all coauthors to register for an ORCID iD and use it as well. Please encourage your coauthors to verify their ORCID iD within the submission system before final acceptance, as unverified ORCID iDs will not appear in the published article. *Only*  the individual author can complete the verification step; PLOS staff *cannot*  verify ORCID iDs on behalf of authors.

We look forward to receiving your revised manuscript.

Kind regards,

Francesca D'Elia, Ph.D.

Academic Editor

PLOS One

Journal Requirements:

When submitting your revision, we need you to address these additional requirements.

1. Please ensure that your manuscript meets PLOS ONE's style requirements, including those for file naming. The PLOS ONE style templates can be found at

https://journals.plos.org/plosone/s/file?id=wjVg/PLOSOne_formatting_sample_main_body.pdf and

https://journals.plos.org/plosone/s/file?id=ba62/PLOSOne_formatting_sample_title_authors_affiliations.pdf

2. Please include your full ethics statement in the ‘Methods’ section of your manuscript file. In your statement, please include the full name of the IRB or ethics committee who approved or waived your study, as well as whether or not you obtained informed written or verbal consent. If consent was waived for your study, please include this information in your statement as well.

3. If the reviewer comments include a recommendation to cite specific previously published works, please review and evaluate these publications to determine whether they are relevant and should be cited. There is no requirement to cite these works unless the editor has indicated otherwise.

4. When completing the data availability statement of the submission form, you indicated that you will make your data available on acceptance. We strongly recommend all authors decide on a data sharing plan before acceptance, as the process can be lengthy and hold up publication timelines. Please note that, though access restrictions are acceptable now, your entire data will need to be made freely accessible if your manuscript is accepted for publication. This policy applies to all data except where public deposition would breach compliance with the protocol approved by your research ethics board. If you are unable to adhere to our open data policy, please kindly revise your statement to explain your reasoning and we will seek the editor's input on an exemption. Please be assured that, once you have provided your new statement, the assessment of your exemption will not hold up the peer review process.

Additional Editor Comments:

Both reviewers agree that the manuscript addresses a relevant topic and offers a valuable multi stakeholder perspective. However, they also identify several substantial issues that require major revision before the manuscript can be reconsidered.

The main concerns relate to:

• Repetition and clarity of writing, particularly in the Introduction and throughout the manuscript.

• Referencing style and accuracy, including missing citations for interpretative claims.

• Methodological transparency, especially regarding sampling procedures, recruitment, saturation, reflexivity, and the rationale for conducting only one focus group per stakeholder group.

• Insufficient detail in the analytic process, including coding procedures, theme development, and strategies to ensure credibility.

• Presentation of qualitative findings, which currently relies heavily on summaries and tables; both reviewers recommend incorporating more direct quotations to allow participants’ voices to emerge.

• Conceptual positioning, where the distinction between qualitative descriptive and phenomenological elements should be clarified.

• Contextualization within the literature, which would benefit from strengthening and reducing redundancy.

• Minor issues, including terminology in tables and refinement of keywords.

Given the extent of these revisions, I invite the authors to thoroughly revise the manuscript by addressing all reviewer comments, improving methodological rigor, and enhancing the presentation and interpretation of qualitative findings. A substantially strengthened version will be required for further consideration.

[Note: HTML markup is below. Please do not edit.]

Reviewers' comments:

Reviewer's Responses to Questions

**Comments to the Author**

1. Is the manuscript technically sound, and do the data support the conclusions?

The manuscript must describe a technically sound piece of scientific research with data that supports the conclusions. Experiments must have been conducted rigorously, with appropriate controls, replication, and sample sizes. The conclusions must be drawn appropriately based on the data presented.

Reviewer #1: Partly

Reviewer #2: Partly

**********

2. Has the statistical analysis been performed appropriately and rigorously?

Reviewer #1: N/A

Reviewer #2: N/A

**********

3. Have the authors made all data underlying the findings in their manuscript fully available?

The PLOS Data policy requires authors to make all data underlying the findings described in their manuscript fully available without restriction, with rare exception (please refer to the Data Availability Statement in the manuscript PDF file). The data should be provided as part of the manuscript or its supporting information, or deposited to a public repository. For example, in addition to summary statistics, the data points behind means, medians and variance measures should be available. If there are restrictions on publicly sharing data—e.g. participant privacy or use of data from a third party—those must be specified.

Reviewer #1: Yes

Reviewer #2: No

**********

4. Is the manuscript presented in an intelligible fashion and written in standard English?

PLOS ONE does not copyedit accepted manuscripts, so the language in submitted articles must be clear, correct, and unambiguous. Any typographical or grammatical errors should be corrected at revision, so please note any specific errors here.

Reviewer #1: Yes

Reviewer #2: Yes

**********

5. Review Comments to the Author

Please use the space provided to explain your answers to the questions above. You may also include additional comments for the author, including concerns about dual publication, research ethics, or publication ethics. (Please upload your review as an attachment if it exceeds 20,000 characters)

Reviewer #1: Journal: PLOS One PONE-D-26-01877

Title: Perceived Academic Achievement and School Commitment in the Context

of School Sports: A Qualitative Study Based on Students’, Teachers’, and

Parents’ Perspectives

Review: 27 February 2026

This article is clearly written and describes each aspect of the research in detail. The writing style is easy to follow. However, there was considerable repetition of information, no doubt the authors were ensuring the reader is clear on where they are in the process. I recommend the authors review their writing to consider removing some of the repetition that is acting as a reminder to the reader.

I felt the research topic was valid and a worthwhile cause to investigate. From a personal perspective I would prefer references to be included in the text as Author and date rather than as a number. This means that as I read the text I know who the referenced authors are and if I am not familiar with them I can go to the reference list to see the named publication. [On this note what is the referencing style for this Journal? I recommend the authors review all references they have made to ensure the style is correct.]

The framework the authors used to examine their data was clear and relevant to their research. The authors were careful to validate their choices and approached the research with a clear mandate of what they sort to achieve. The process of using a pilot study added strength to this research.

Given that this was qualitative research I would have like to know what motivated the researchers to participate. Where were they in the narrative?

The following is an excerpt from the research. Be mindful of referencing all claims to information or own it as your own. I am unsure who thinks or says this.

Although school sports are frequently promoted as mechanisms for enhancing discipline,

belonging, and academic motivation, there remains limited qualitative evidence explaining how

these outcomes are perceived by students, teachers, and parents within the same educational

context. In particular, descriptive research that systematically documents participants’ views on

both the perceived benefits and challenges of school sports remains scarce. P12 line 131 to 135.

My advice given this example is for the authors to check that key information such as this quote is acknowledged in some way. Review of the entire article will ensure that their research retains validity in terms of who says what.

P13 Table 1: What is Athleticism? Or should the Sport have been Athletics

P14 Table 3: What is meant by the column Branch? Do you mean the parents area of expertise or study?

The authors recorded the questions for the three focus groups. It was interesting to read them but in a journal article I feel this detail is not necessary… unless the authors are using the questions to highlight the responses. This brings me to my disappointment at not being able to read something of what the research participants had said. Instead the authors have used tables to record a limited number of responses which in some way makes the research more quantitative than qualitative. The point of using the methods the authors used to obtain data was to enable the voices of the participants to be heard. Unfortunately this was taken away. Rather the authors have chosen to summarise and paraphrase their participants voices. I recommend that the authors think differently in how they have presented their research and allow the voices, the questions and the summaries to speak from the participants. This would require some reworking of your manuscript but in my view would strengthen the findings. You would still be able to utilise some of the writing and explanations in the discussions and conclusion. I commend you on clearly establishing the strengths and limitations of your research and the recommendations that have emerged from your thinking.

In considering restructuring your article, please think about including the literature about sport in schools and how it is viewed to contextualise your research. I think you have done this to a certain extent but in my view this could be strengthened. I note that some of this information is contained in your discussion. What you have here is a good narrative about school sport participation and how it is viewed and experienced. Let the voices of the participants tell their story while you the authors and researchers write the commentary that unpacks the findings.

I think this is a good article but suggest that if you were to follow some recommendations you could make it better because it is a hot topic and the information you tell is useful to other practitioners even if they are not in Turkey.

Reviewer #2: Firstly, I would like to express my gratitude for the opportunity to review this article and offer my opinion. The considerations presented here are intended to enhance the overall quality and clarity of the manuscript.

The manuscript addresses a relevant and timely topic concerning the relationship between school sports, academic achievement, and school commitment from a multi-stakeholder perspective. Although the research question is not entirely novel, the inclusion of students, teachers, and parents represents a valuable contribution. However, the manuscript presents several methodological and analytical limitations that require substantial revision. With improvements, the study may contribute to the existing literature, particularly as an exploratory qualitative investigation highlighting the contextual and perceived effects of school sports within a specific educational setting.

Specific Comments

Title: The title is clear, well-structured, and consistent with the study’s aim and design.

Abstract

The abstract is well written and provides a clear overview of the study. However, it is recommended to revise the keywords to avoid repetition of terms already present in the title. Using more diverse keywords may improve the discoverability of the article.

Introduction

The Introduction is comprehensive and theoretically grounded. It clearly frames the research problem and situates the study within the literature. However, the section is somewhat repetitive in places, particularly regarding the definition of school commitment and the role of sports. Additionally, the epistemological positioning of the study (qualitative descriptive vs. phenomenological elements) should be clarified to avoid conceptual ambiguity.

Materials and Methods

Sampling: The manuscript states that criterion sampling was used, but it is unclear how participants were recruited, approached, or whether any refused to participate. These details are essential for evaluating potential biases.

Sample Size: Only 6 participants per stakeholder group (18 total) were included. While acceptable for qualitative research, the study does not discuss how data saturation was assessed or justified the small sample.

Focus Groups: Only one focus group per stakeholder category was conducted. It is recommended to justify why a single focus group was sufficient to capture diverse perspectives.

Data Collection Tool: The interview guide is described, but there is no example of how questions were refined during piloting or how probes were applied in practice. Including specific illustrative questions and examples of follow-up prompts would strengthen transparency.

The manuscript mentions that the first author moderated the focus groups but provides little discussion of potential biases or how they were managed. Reflexivity is critical in qualitative studies to assess credibility.

Data Analysis:

The coding process is not sufficiently transparent. Were codes double-checked, software used, or inter-coder agreement calculated?

While inductive content analysis is mentioned, the manuscript does not describe how categories were grouped into higher-order themes. Including a coding tree or matrix would help readers understand theme derivation.

The analysis largely remains descriptive, with limited interpretation of relationships between stakeholder perspectives.

Discussion

The Discussion reiterates results but does not sufficiently interpret them in context.

For example:Divergent parent and student perceptions regarding academic risks are described, but why these divergences exist and how they relate to cultural, institutional, or developmental factors is underexplored.

Teachers’ mediating role is mentioned, but specific mechanisms or examples (e.g., feedback, monitoring, scheduling adjustments) should be detailed.

Contextual limitations (single-school, Turkish setting) are acknowledged, but their implications for transferability and interpretation should be analyzed more deeply.

The multi-stakeholder approach is a strength, yet the discussion does not clearly explain what unique insights it provides beyond existing literature.

Potential challenges like time management and academic fatigue are noted but the discussion could link these findings to prior studies or theoretical frameworks, clarifying whether they are consistent or novel.

Conclusion

The conclusions are consistent with the study’s aims and findings. However, given the exploratory nature of the research, it is recommended to emphasize more clearly that the results are context-specific and not generalizable.

References

The references are generally appropriate and relevant, although some sections could benefit from the inclusion of more recent studies.

**********

6. PLOS authors have the option to publish the peer review history of their article (what does this mean?). If published, this will include your full peer review and any attached files.

If you choose “no”, your identity will remain anonymous but your review may still be made public.

**Do you want your identity to be public for this peer review?**  For information about this choice, including consent withdrawal, please see our Privacy Policy.

Reviewer #1: No

Reviewer #2: No

**********

[NOTE: If reviewer comments were submitted as an attachment file, they will be attached to this email and accessible via the submission site. Please log into your account, locate the manuscript record, and check for the action link "View Attachments". If this link does not appear, there are no attachment files.]

To ensure your figures meet our technical requirements, please review our figure guidelines: https://journals.plos.org/plosone/s/figures

You may also use PLOS’s free figure tool, NAAS, to help you prepare publication quality figures: https://journals.plos.org/plosone/s/figures#loc-tools-for-figure-preparation.

NAAS will assess whether your figures meet our technical requirements by comparing each figure against our figure specifications.

# Author comment: Author response to Decision Letter 1

**DOI:** 10.1371/journal.pone.0351877.r002

6 May 2026

Under the “Respond to Reviewers” section in the journal panel, we would like to state that responses to all reviewer comments have been provided. The responses have also been incorporated into the revised manuscript through appropriate revisions. In addition, a “Revised Manuscript with Track Changes” file has been prepared and submitted.

Due to our academic requirements, we kindly hope for your support in advancing the manuscript to the publication stage.

Thank you for your consideration, and we wish you a healthy and productive day.

Attachment Submitted filename: Response to Reviewers.docx

# Peer review: Decision Letter 1

**DOI:** 10.1371/journal.pone.0351877.r003

**Authors:** Francesca D'Elia

2 Jun 2026

Okul Sporları Bağlamında Algılanan Akademik Başarı ve Okula Bağlılık: Öğrencilerin, Öğretmenlerin ve Velilerin Bakış Açılarına Dayalı Niteliksel Bir Çalışma

PONE-D-26-01877R1

Dear Dr. Taş,

We’re pleased to inform you that your manuscript has been judged scientifically suitable for publication and will be formally accepted for publication once it meets all outstanding technical requirements.

Within one week, you’ll receive an e-mail detailing the required amendments. When these have been addressed, you’ll receive a formal acceptance letter and your manuscript will be scheduled for publication.

An invoice will be generated when your article is formally accepted. Please note, if your institution has a publishing partnership with PLOS and your article meets the relevant criteria, all or part of your publication costs will be covered. Please make sure your user information is up-to-date by logging into Editorial Manager at Editorial Manager® and clicking the ‘Update My Information' link at the top of the page. For questions related to billing, please contact billing support.

If your institution or institutions have a press office, please notify them about your upcoming paper to help maximize its impact. If they’ll be preparing press materials, please inform our press team as soon as possible -- no later than 48 hours after receiving the formal acceptance. Your manuscript will remain under strict press embargo until 2 pm Eastern Time on the date of publication. For more information, please contact onepress@plos.org.

Kind regards,

Francesca D'Elia, Ph.D.

Academic Editor

PLOS One

Additional Editor Comments (optional):

Thank you for the revised manuscript. Both reviewers now support acceptance. Before final approval, please address the remaining minor corrections noted by Reviewer 1:

- Replace “Profession” with a more appropriate term (e.g., “Sports Code”).

- Replace “Branch” with “Curriculum” in the table.

- Verify the age reported for Parent 18, which appears inconsistent.

Once these small adjustments are made, the manuscript will be ready for acceptance.

Reviewers' comments:

Reviewer's Responses to Questions

**Comments to the Author**

1. If the authors have adequately addressed your comments raised in a previous round of review and you feel that this manuscript is now acceptable for publication, you may indicate that here to bypass the “Comments to the Author” section, enter your conflict of interest statement in the “Confidential to Editor” section, and submit your "Accept" recommendation.

Reviewer #1: All comments have been addressed

Reviewer #2: All comments have been addressed

**********

2. Is the manuscript technically sound, and do the data support the conclusions?

The manuscript must describe a technically sound piece of scientific research with data that supports the conclusions. Experiments must have been conducted rigorously, with appropriate controls, replication, and sample sizes. The conclusions must be drawn appropriately based on the data presented.

Reviewer #1: Yes

Reviewer #2: Yes

**********

3. Has the statistical analysis been performed appropriately and rigorously?

Reviewer #1: N/A

Reviewer #2: N/A

**********

4. Have the authors made all data underlying the findings in their manuscript fully available?

The PLOS Data policy requires authors to make all data underlying the findings described in their manuscript fully available without restriction, with rare exception (please refer to the Data Availability Statement in the manuscript PDF file). The data should be provided as part of the manuscript or its supporting information, or deposited to a public repository. For example, in addition to summary statistics, the data points behind means, medians and variance measures should be available. If there are restrictions on publicly sharing data—e.g. participant privacy or use of data from a third party—those must be specified.

Reviewer #1: Yes

Reviewer #2: Yes

**********

5. Is the manuscript presented in an intelligible fashion and written in standard English?

PLOS ONE does not copyedit accepted manuscripts, so the language in submitted articles must be clear, correct, and unambiguous. Any typographical or grammatical errors should be corrected at revision, so please note any specific errors here.

Reviewer #1: Yes

Reviewer #2: Yes

**********

6. Review Comments to the Author

Please use the space provided to explain your answers to the questions above. You may also include additional comments for the author, including concerns about dual publication, research ethics, or publication ethics. (Please upload your review as an attachment if it exceeds 20,000 characters)

Reviewer #1: Review 2: 29 May 2026

In reviewing this article for the second time I felt more comfortable with the format and the recording of events. The Tables were constructed with clarity. I like the way the author’s included the voices of their participants more fully than previously.

I realised the description of the research process provides a very effect means to show what was done and how. It will provide useful information for others conducting qualitative research to follow because it explains and answers questions and problems that may arise. This kind of information is important for future researchers as it is very much a ‘hands on’ account.

I have noted a few errors or points to address below. In particular, there are a couple of English words that have been chosen as headings in the tables that I do not think make sense. I have made suggestions. I would like to say the English version is well written with few errors.

This article is ready for publication. Well done.

Some Corrections.

P 8 L180 Title Profession is not the correct English. Perhaps a phrase such as Sports Code

P 9 Table Branch I think a better word would be Curriculum – being the area that these people participated or majored in.

P 9 Table Parent 18 is aged 26 I think this is an incorrect age. I assume each parent is paired with a student in the study.

Reviewer #2: (No Response)

**********

7. PLOS authors have the option to publish the peer review history of their article (what does this mean?). If published, this will include your full peer review and any attached files.

If you choose “no”, your identity will remain anonymous but your review may still be made public.

**Do you want your identity to be public for this peer review?**  For information about this choice, including consent withdrawal, please see our Privacy Policy.

Reviewer #1: **Yes:**  Dr Maureen Legge

Reviewer #2: No

**********

# Editor report: Acceptance letter

**DOI:** 10.1371/journal.pone.0351877.r004

**Authors:** Francesca D'Elia

PONE-D-26-01877R1

PLOS One

Dear Dr. Taş,

I'm pleased to inform you that your manuscript has been deemed suitable for publication in PLOS One. Congratulations! Your manuscript is now being handed over to our production team.

At this stage, our production department will prepare your paper for publication. This includes ensuring the following:

* All references, tables, and figures are properly cited

* All relevant supporting information is included in the manuscript submission,

* There are no issues that prevent the paper from being properly typeset

You will receive further instructions from the production team, including instructions on how to review your proof when it is ready. Please keep in mind that we are working through a large volume of accepted articles, so please give us a few days to review your paper and let you know the next and final steps.

Lastly, if your institution or institutions have a press office, please let them know about your upcoming paper now to help maximize its impact. If they'll be preparing press materials, please inform our press team within the next 48 hours. Your manuscript will remain under strict press embargo until 2 pm Eastern Time on the date of publication. For more information, please contact onepress@plos.org.

You will receive an invoice from PLOS for your publication fee after your manuscript has reached the completed accept phase. If you receive an email requesting payment before acceptance or for any other service, this may be a phishing scheme. Learn how to identify phishing emails and protect your accounts at https://explore.plos.org/phishing.

If we can help with anything else, please email us at customercare@plos.org.

Thank you for submitting your work to PLOS ONE and supporting open access.

Kind regards,

PLOS ONE Editorial Office Staff

on behalf of

Dr. Francesca D'Elia

Academic Editor

PLOS One
